# Diabetic yoga protocol improves glycemic, anthropometric and lipid levels in high risk individuals for diabetes: a randomized controlled trial from Northern India

**DOI:** 10.1186/s13098-021-00761-1

**Published:** 2021-12-23

**Authors:** Navneet Kaur, Vijaya Majumdar, Raghuram Nagarathna, Neeru Malik, Akshay Anand, Hongasandra Ramarao Nagendra

**Affiliations:** 1 Department of Physical Education, Panjab University, Chandigarh, 160014 India; 2 Division of Life Sciences, Swami Vivekananda Yoga Anusandhana Samsathana, Bengaluru, Karnataka 560106 India; 3 Dev Samaj College of Education, Sector 36B, Chandigarh, 160036 India; 4 Department of Neurology, Neuroscience Research Lab, Postgraduate Institute of Medical Education and Research, Chandigarh, 160012 India

**Keywords:** Diabetic yoga protocol, Indian diabetes risk score, Glycated hemoglobin, Diabetes, Prediabetes

## Abstract

**Purpose:**

To study the effectiveness of diabetic yoga protocol (DYP) against management of cardiovascular risk profile in a high-risk community for diabetes, from Chandigarh, India.

**Methods:**

The study was a randomized controlled trial, conducted as a sub study of the Pan India trial *Niyantrita Madhumeha Bharath (NMB)*. The cohort was identified through the Indian Diabetes Risk Scoring (IDRS) (≥ 60) and a total of 184 individuals were randomized into intervention (n = 91) and control groups (n = 93). The DYP group underwent the specific DYP training whereas the control group followed their daily regimen. The study outcomes included changes in glycemic and lipid profile. Analysis was done under intent-to-treat principle.

**Results:**

The 3 months DYP practice showed diverse results showing glycemic and lipid profile of the high risk individuals. Three months of DYP intervention was found to significantly reduce the levels of post-prandial glucose levels (p = 0.035) and LDL-c levels (p = 0.014) and waist circumference (P = 0.001).

**Conclusion:**

The findings indicate that the DYP intervention could improve the metabolic status of the high-diabetes-risk individuals with respect to their glucose tolerance and lipid levels, partially explained by the reduction in abdominal obesity. The study highlights the potential role of yoga intervention in real time improvement of cardiovascular profile in a high diabetes risk cohort.

*Trial registration:* CTRI, CTRI/2018/03/012804. Registered 01 March 2018—Retrospectively registered, http://www.ctri.nic.in/CTRI/2018/03/012804.

## Introduction

The rise of diabetes in the developing world poses a threat to meager health budgets. Owing to the strong association between various morbidity and mortality outcomes as complications of this dreaded disease, early detection of diabetes risk through non-invasive parameters is a primary requisite. Observational studies show that the risk reduction for diabetes can be decreased by 58% or 63–65% if risk factors could be controlled [1, 2]. Many argue that such experimental strategies for the possible halting of conversion of prediabetes into diabetes must continue to include pharmacological interventions even though the rates have not been compared [3]. Identification of individuals at increased risk for the disease with invasive measurements of fasting and post challenge (postprandial) blood glucose are costly and time consuming. Hence, it has been advocated that the realistic prevention of diabetes should identify high-risk subjects with the use of the non-invasive risk scores [4]. Such studies should also target subjects with normoglycemia and prevent their progression to poor glycemic status [4].

Yoga plays a promising role in minimizing the risk of Diabetes for high-risk individuals with prediabetes [5, 6]. It reduces body weight, glucose, and lipid levels, though, most of these studies comply with the guidelines of randomized controlled trials adhered to the CONSORT statements [7–11] whereas majority of studies have not reported as per CONSORT statements [12–15]. Several review of published studies, in people with diabetes and prediabetes, have concluded that the practice of yoga may reduce insulin resistance and related cardiovascular disease (CVD) risk factors and improve clinical outcomes [16]. Specifically, reports suggest that a yoga-based lifestyle intervention reduces body weight, glucose and lipid levels that should reduce diabetes risk. Keeping in view the high transition rates of diabetes in India, we selected a high-risk cohort from Chandigarh, one of the most affluent Union Territories of India with highest reported prevalence of diabetes in order to establish the efficacy of yoga to alleviate the cardiovascular disease. Indian Diabetes Risk Score (IDRS), specific for Indian ethnicity a validated tool was used for identification of the high-risk population [17]. We developed a national consensus ‘Diabetes Yoga protocol’ based on published reports and classical literature with an aim to stimulate weight reduction by combination of postures and meditation techniques [18, 19]. Additionally, cardiometabolic risk reduction has also been recognized as one of the potential outcomes of yoga-based interventions [20]. Yoga has been shown to be regulating the risk parameters of diabetes, waist circumference (WC), body mass index (BMI), oxidative stress, fasting blood sugar (FBS) and systolic blood pressure (SBP) respectively [21]. Hence, in this study we tested the efficacy of diabetic yoga protocol (DYP) on alleviation of glycemic and lipid imbalances in individuals at high risk of diabetes.

## Materials and methods

### Study population

Under the multi-region survey of *Niyantarita Maduhmeha Bharat* (NMB-2017) a door-to-door screening was carried out for the identification of high risk individuals among the population of Chandigarh (U.T) and Panchkula (District in Haryana state) on the basis of Indian Diabetes Risk Score (IDRS). The data collection was carried out by well trained yoga volunteers for diabetes management (YVDMs). Written informed consents were taken from every subject during door to door screening as well as at the time of registration. All the experimental protocol, methods and procedures were approved by Ethics committee of Indian Yoga Association (IYA) (ID: RES/IEC-IYA/001). All experiments methods and procedures were carried out in accordance with relevant guidelines and regulations of ethics committee. The study was registered at clinical trial registry of India, CTRI/2018/03/012804 (dated: 01/03/2018).

### Study design

**Fig. 1:**
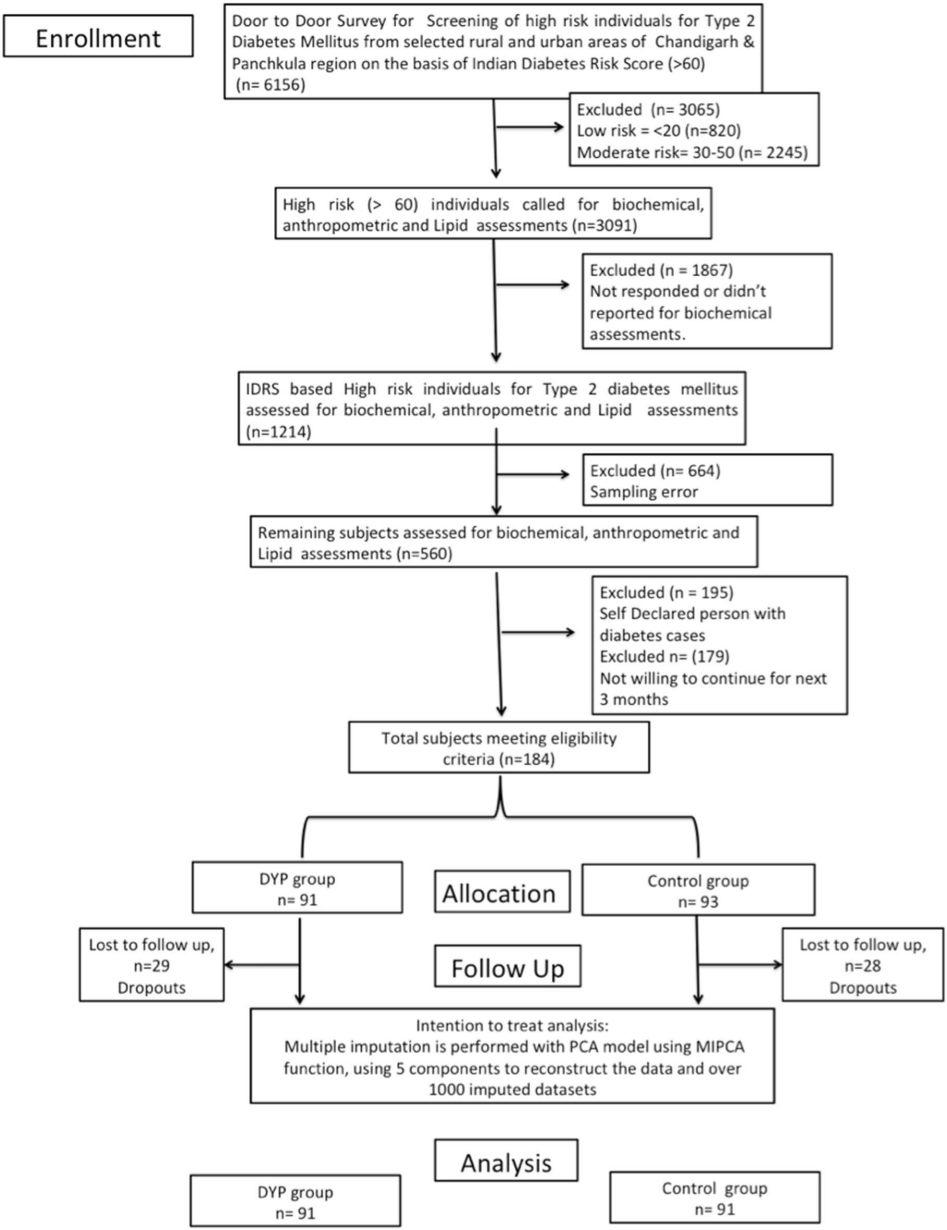
Flowchart of study design. *PCA*  principal component analysis, *MIPCA* multiple imputations with PCA

The present study is the two-armed randomized controlled trial conducted in the population of Chandigarh and Panchkula regions of northern India. Indian Diabetes Risk Score (IDRS) was used for detection of high risk (≥ 60 score) individuals from the study. Self-declared diabetics and low (< 30 score) and moderate [between 30–50 score] risk individuals were excluded from the study. As evident from the flow of patients presented in the flowchart, out of 1214 eligible subjects, there was approximately 50% loss of sample data due to error in the sampling. Further out of 564, we had to exclude as they were self-declared patients with diabetes and did not further participate in the study. However, this led to final participation of only 184 subjects in the study and allocation of these subjects diminishing the random selection of the study cohort. A cohort of high diabetes-risk cohort consisting of n = 184 participants was randomized into the interventional and control groups (n = 91:93). After excluding the dropouts from the study, based on CONSORT guidelines, the remaining subjects in the DYP and control group were further assessed for selected anthropometric, glycemic and lipid parameters. The intervention group was given the Diabetic Yoga Protocol for three months and control group continued with their daily routine activities. The detailed categorization of the samples is shown in Fig. 1. The control group was waitlisted for yoga.

### Randomization

Simple randomization technique was used to allocate participants into the intervention and the control groups. An independent statistician generated a computer-generated random number sequence and the sequence was given to an external staff who had no involvement in the study procedures. The participants were allocated their consecutive numbers, after baseline measurements. Blinding of the participants was not possible due to the nature of the intervention. However, the outcome assessors were blinded.

### Risk assessment

To identify the individuals at high-risk of diabetes, Indian Diabetes Risk Score (IDRS) was administered as proposed by Mohan et al. [22]. It consisted of two unmodifiable (i.e. age, and family history) and two modifiable (physical activity and waist circumference) risk factors for diabetes, which can predict the level of risk for the development of diabetes in the community. The IDRS is one of the easily accessible and budget friendly questionnaire to be administered. The aggregate score of the unmodifiable and modifiable risk used to probe the level of risk among the population (i.e. High risk > 60, Moderate risk-30–50, Low risk < 30).

### Sample size

Sample size estimation for the main Pan India study was focused for prediabetes subjects [23]. However, for the present pilot scale study we calculated sample size assuming a small effect size 0.3 [5] of DYP vs waitlist control 0.25, α = 0.80 as 180 (n = 90:90). Further, assuming an attrition rate of 20%, the final sample size was n = 220.

### Study outcomes

Changes in the glycemic and other metabolic variables (anthropometric and lipid) over 3 months were documented. The fasting blood sample was withdrawn. For glucose analysis, fasting samples for 10–12 h were taken early in the morning for the estimation of FBS and afterwards 75 g glucose was given to the participants. The blood sampling was repeated after 2 h. for estimation of OGTT.

### Biochemical analysis

For the estimation of biochemical parameters viz. FBS (Fasting Blood Sugar, Rxl-Max 500), OGTT (Oral Glucose Tolerance Test), HbA1c (Bio-Rad D-10), Triglycerides, Cholesterol, HDL, LDL, Chol/HDL ratio, HDL/LDL ratio (Rxl-Max 500) and VLDL about 9 ml of blood was drawn and analyzed by phlebotomist of Sisco Research Laboratories (SRL) of Chandigarh. Anthropometric measurements were also obtained (i.e. height, weight, waist circumference) by trained researcher. The waist circumference (WC) was reported in centimeters. The BMI was obtained by using the formula (weight in kg/height (meter)^2^).

#### Interventions

The study protocol consisted of Diabetic Yoga Protocol (DYP) approved by the Ministry of AYUSH and Quality Council of India as shown in Table 1. This is the first protocol to be made specifically for the prediabetics and diabetics. The complete sequence of prayer, yogic postures, breathing and meditative techniques, along with specified time, was shown in previously published paper [24]. The Yogic practices were performed for 3 months for 60 min. Certified yoga instructors took the yoga classes and they recorded regular attendance. Randomization was done through a computer-generated list of random numbers and allocation was concealed to the participants until the completion of the baseline assessment.

**Table 1:** Diabetic yoga protocol (DYP)

S. No.	Name of practice	Duration (min)
1	Starting prayer: Asatoma Sat Gamaya	2
2	Preparatory Sukshma Vyayamas and Shithililarna Practices 1. *Urdhavahastashvasan(Hand stretching breathing 3 rounds at 90°, 135° and 180*^*o*^* each)* 2. *Kati-Shakti Vikasaka* (3 rounds) *a) Forward and Backward Bending b) Twisting* 3. Sarvangapushti (3 rounds clockwise, 3 rounds anticlockwise)	6
3	Surya Namaskara (SN) 10 step fast *Surya Namaskara* 6 rounds 12 step slow *Surya Namaskara* 1 round Modified version Chair SN 7 rounds	9
4	Asanas (1 min per Asana) 1 *Standing Position (1 min per Asana)* Trikonasana, Parvritta Trikonasana, Prasarita Padhastasana 2 *Supine Position* Jatara Parivartanasana, Pawanmuktasana, Viparitakarani 3 *Prone Position* Bhujangasana, Dharuasana followed by Pawanmuktasana 4 *Sitting Position* Mandukasana, Vakrasana/ Ardhamatsayendrasana, Paschimatanasana, Ardha Ushtrasana At the end, relaxation with abdominal breathing in supine position (vishranti), 10–15 rounds (2 min)	15
5	Kriya a. *Agnisara*:1 min b. *Kapalabhati(@60 breaths per minute for 1 min followed by rest for 1 min)*	3
6	Pranayama *Nadishuddhi *(*for 6 min, with antarkumbhak and jalandhar bandh for 2 s*) *Bhamari 3 min*	9
7	Meditation (for Stress, for deep relaxation and silencing of mind) Cyclic Meditation	15
8	Resolve (I am Completely Healthy)	1
9	Closing Prayer: Sarvebhavantu Sukhina…………	1
	Total duration	60

### Statistical analysis

For the analysis of data SPSS for Windows (version 22; IBM SPSS Inc., Chicago IL) 0 and R statistical package were used. The normality of data was analyzed using Kolmogorov–Smirnov test. The paired t-test was used to estimate the Baseline and posttest differences of DYP, and control group and the significant level was set at ≤ 0.05. The trial outcomes were analyzed according to the intention-to-treat principle; hence multiple imputation was carried for the missing variables accounting for the loss to follow up. We used absolute change (time and treatment interaction), to estimate intervention effects refers to the difference in the outcome of the intervention and control over different time-points of assessment. Absolute change was determined as follows: absolute change = [(intervention group follow-up) – (intervention group baseline)] – [(control group follow-up) – (control group baseline)]. The percentage change, also called the relative change was determined as relative change = (absolute change / intervention group baseline) × 100%. To evaluate the influence of missing data, we applied multiple imputations to the data using missMDA R package (v1.13) based on the principal component analysis method [25] from the package, using 5 components to reconstruct the data and over 1000 imputed datasets. One-way multivariate analysis of covariance (MANCOVA) was conducted to compare the effects of the DYP with control group glycemic and metabolic measures, while controlling for the age, gender and baseline values of the covariates.

## Results

### Baseline characteristics

The data used in this study was collected in (NMB-2017) the northern region of India i.e. Chandigarh and Panchkula. The age range of participants was 3–70 years; [mean age 48.51 (SD 10.08) years]with baseline characteristics of the yoga and control groups as shown in Table 2. Mean HbA1c of the high-risk cohort was 5.64% (0.38), mean FBS was 97.13 mg/dl (SD 11.10), and mean PPBS were 108.40 mg/dl (SD 28.79). Distributions of age and gender was similar between the intervention and the control groups. The IDRS and anthropometric values were also similarly distributed between the groups. Overall, there was no significant difference in the distribution of demographic, anthropometric, or biochemical parameters between the DYP and the control groups at the baseline.

**Table 2:** Baseline characteristics of the participants in the intervention and control group

Characteristics	DYP Group N = 91	Control group N = 93	P value
Gender			
Male, n (%)	19 (20.88)	30 (32.26)	0.096
Age (years)	47.77 (9.59)	49.24 (10.53)	0.323
Weight, Kg	70.93 (10.90)	70.80 (12.44)	0.936
Waist circumference, cm	99.34 (9.05)	99.72 (9.05)	0.794
BMI, Kg/m^2^	28.59 (5.75)	28.53 (5.01)	0.949
IDRS	74.07 (10.43)	75.27 (9.95)	0.425
Biochemical variables			
FBG, mg/dl	96.89 (9.95)	97.36 (12.20)	0.776
PPBG, mg/dl	102.88 (21.91)	113.78 (33.47)	0.012*
HbA1c (%)	5.61 (0.38)	5.66 (0.38)	0.400
Total cholesterol mg/dl	186.88 (37.64)	179.98 (34.98)	0.199
Triglycerides, mg/dl	131.93 (68.59)	138.44 (68.89)	0.522
HDL-c, mg/dl	47.76 (9.16)	48.33 (17.43)	0.780
LDL-c, mg/dl	112.75 (31.02)	104.38 (31.70)	0.072
VLDL, mg/dl	26.39 (13.72)	28.00 (13.50)	0.423

Continuous variables are represented as mean (SD) and compared using independent t-test. Categorical variables are represented as number (percentages) and compared using chi-square test. P value < 0.05 were considered significant. *FBS* fasting blood sugar, *PPBG* postprandial blood glucose, *HbA1c* glycated hemoglobin, *HDL-c* high density lipid-cholesterol, *LDL-c* low density lipid-cholesterol, *VLDL* very low density lipid-cholesterol, *IDRS* Indian diabetes risk score

When analyzed by multivariate analysis of covariance (MANCOVA), adjusting for age, gender and status of diabetes/prediabetes/normoglycemia, and baseline values of the covariates, yoga intervention was found to have significant influence on few cardinal parameters related to glycemic control (PPBS), and lipid control (LDL-C) as shown in Table 3. We also observed a significant influence of DPP on waist circumference reduction [relative changes, − 1.94%. Compared to the control, DYP also resulted in significant reductions in LDL-C and, − 0.16% and − 2.81%, for LDL-Cholesterol and post-prandial blood glucose levels from baseline to 3 months [absolute changes, − 0.18% and − 3.08%, respectively and relative changes, − 0.16% and − 2.81%, respectively].

**Table 3:** Comparative assessment of influence of DYP on biochemical and weight related variables with the control group

Variables	Baseline mean (SD)	After 3 months mean (SD)	Absolute change	Relative change	P value	Partial η2
Waist circumference (cm)						
DYP	99.34 (9.05)	98.14 (6.88)	− 1.93	− 1.94	0.032	0.029
Control	99.72 (9.05)	100.25 (7.72)				
BMI, kg/m2						
DYP	28.59 (5.75)	28.00 (6.84)	− 0.4	− 1.40	0.622	0.002
Control	28.53 (5.01)	28.34 (4.98)				
Weight, Kg						
DYP	70.93 (10.90)	69.04 (9.13)	− 1.04	− 1.47	0.397	0.005
Control	70.80 (12.44)	69.95 (10.44)				
Postprandial blood glucose, mg/dl						
DYP	102.88 (21.91)	118.32 (29.89)	− 1.51	− 1.47	0.006	0.046
Control	113.78 (33.47)	130.73 (36.98)				
Fasting blood glucose, mg/dl						
DYP	96.89 (9.95)	99.82 (9.49)	1.44	1.49	0.287	0.007
Control	97.36 (12.20)	98.85 (9.26)				
HBA1c (%)						
DYP	5.61 (0.38)	5.61 (0.39)	− 0.02	− 0.36	0.077	0.020
Control	5.66 (0.38)	5.68 (0.38)				
Total Cholesterol, mg/dl						
DYP	186.88(37.64)	189.01 (25.64)	− 0.4	− 0.21	0.130	0.014
Control	179.98 (34.98)	182.51(20.82)				
Triglycerides, TG, mg/dl						
DYP	131.93 (68.59)	148.14 (54.92)	− 13.98	− 10.60	0.138	0.014
Control	138.44 (68.89)	168.63 (75.06)				
HDL-C, mg/dl						
DYP	47.76 (9.16)	47.01 (9.16)	2.2	4.61	0.097	0.017
Control	48.33 (17.43)	45.38 (12.57)				
LDL-C, mg/dl						
DYP	112.75 (31.02)	103.39 (21.44)	− 17.56	− 15.57	0.044*	0.025
Control	104.38 (31.70)	112.58 (21.99)				
VLDL, mg/dl						
DYP	26.39 (13.72)	28.85 (10.47)	− 1.23	− 4.66	0.229	0.009
Control	28.00 (13.50)	31.69 (10.57)				

Absolute change = [(intervention group follow-up) – (intervention group baseline)] – [(control group follow-up) – (control group baseline)]. Relative change = (absolute change / intervention group baseline) × 100%; p value for difference between the intervention and the control groups by MANCOVA adjusting for age, gender, status of diabetes/prediabetes/normoglycemia baseline values of glycemic and lipid variables, length of time having had prior exposure of yoga

## Discussion

We examined the effect of Diabetic Yoga Protocol on baseline and post (3 months) levels of HbA1c and other glycemic (OGTT and FBS), Lipid (Total cholesterol, triglycerides, HDL-c, LDL-c, and VLDL-c, CDL/HDL, LDL/HDL) and anthropometric parameters (BMI). In the present study, we show the efficacy of DYP in substantial improvement in the waist circumference in a high-risk diabetes population from Chandigarh (relative change of 1.94 cm). We could also demonstrate a significant decline in the worsening of post prandial glucose levels with yoga intervention as compared to the wait-list control group (relative change of 2.82 mg/ml). However, for LDL-c levels, there were clinically significant improvements by 0.16 units. Notably, over 3 months study duration there was an overall increase in the levels of total cholesterol, triglyceride and VLDL means in the study cohort, while HDL levels had decreased. In particular TG levels have gone from normal range to mildly high (> 150 mg/dl) [26] which draws our attention towards accelerated pace of metabolic dysfunction in the high risk population. These findings comply with Chandigarh being an affluent union territory of India with high per-capita GDP and has been documented to have highest prevalence of diabetes 13.6%, 12.8–15·2 as compared to other Indian states [27]. As mentioned above, there was a significant influence of DYP on the waist circumference, one of the two important modifiable parameters of Indian Diabetes Risk Score [17]. The relevance of WC reduction in context of reduced risk of CVD is well established; a 1 cm increase in WC has been associated with a 2% increase in the relative risk of future CVD [28]. The visceral adipose tissue is a primary source of cytokine production and insulin resistance (IR) [29]. Given the higher susceptibility towards visceral fat accumulation and insulin resistance in Asian populations as compared to their Caucasian counterparts, the observed influence of DYP on WC is of particular relevance to the metabolically obese phenotype of Asian Indians [30].

In relation to the glucose metabolism, we could also demonstrate a significant decline in the worsening of post prandial glucose levels with DYP as compared to the wait-list control group (relative change = − 2.81%, P < 0.05); however, no significant influence could be established for fasting blood glucose concentration. These findings could be justified by the phenotypic differences underlying fasting and post-challenge hyperglycemia that represent distinct natural histories in the evolution of type 2 diabetes [31]. Postprandial glucose disposal is the primary pathogenic manifestation in impaired glucose tolerance (IGT), and impaired fasting glucose (IFG) merely signifies an abnormal glucose set point [31, 32]. Our relevance of the study findings is further underlined by the previous results wherein PPG has been reported to contribute more than FBS to overall hyperglycemia and its control was found essential either to decrease or to obtain HbA1c goals of < 7 [33]. Several epidemiological studies have suggested that increased glycemic exposure, especially post challenge or postprandial hyperglycemia, is an independent risk factor for macrovascular disease with no apparent upper or lower threshold. Our results indicate a significant influence of yoga on glycemic control integrating postprandial glycemic alterations in the high diabetes risk group. Since in the present study the high-risk cohort was selected through A1c based diagnosis, and IGT was not a primary manifestation in the cohort, hence, the overall improvement in postprandial glucose should be specifically tested in an IGT cohort. The findings of the current study with a 3-month intervention of yoga on postprandial measures of glucose at-risk population deserves clinical attention. Increase in the glucose concentration even in the prediabetes stage, manifests as a chronic inflammatory condition and predisposes an individual to the risk of pathogenic infections [32, 34, 35].

The simultaneous reduction in waist circumference observed in the cohort, is also consistent with the observation of an association between abdominal obesity and the risk of IGT. Based on a significant association between IGT and CVD risk [32, 33, 36], we note a significant improvement in lipid concentrations [LDL-c] by the DYP protocol as compared to the control group. These results are consistent with the previously reported overall beneficial effect of yoga in the management of hyperlipidemia [36]. These results need validation at larger scale and to ascertain the mechanistic insights into the action of yoga, the indices of monocyte chemotaxis, endothelial inflammation, oxidation, nitric oxide production, and thrombosis should also be explored [37], including animal models, invitro systems and other approaches [38–44].

The findings of the present study indicate that identification of high-risk group through IDRS and consequent intervention of Yoga based lifestyle protocol could be an effective strategy to combat the metabolic perturbations associated with diabetes, whose co-morbidity is also being reported to be associated with increasing vulnerability to the emerging viral pandemic of COVID-19. Lifestyle interventions are reported to reduce the risk of Type 2 diabetes in high-risk individuals after mid and long-term follow-up. Information on determinants of intervention outcome, adherence and the mechanisms underlying diabetes progression are valuable for a more targeted implementation. Weight loss is a major contributor in the prevention and management of type 2 diabetes. In many of the earlier lifestyle intervention group of the DPP, weight loss was the dominant predictor of reduced diabetes risk, with a 16% reduction observed for every kilogram of weight loss during the 3.2-year follow-up [45]. Though we failed to observe a significant weight loss over 3 months of DYP intervention, the significant reductions in WC indicate the plausibility of significant weight loss on longer interventions and follow ups.

Whether Yoga alters the conversion of prediabetics into healthy status and if it helps in maintenance of glycemic index can be assessed by longitudinal studies. There was a significant improvement in the glycemic status of the high risk population at administration of DYP. The analysis shows the aptness of Diabetic protocol which is apparently superior to previous studies where no standardized protocols were used for intervention [46, 47]. The findings suggest that there is potential of DYP to manage glucose levels in diabetes patients if public intervention is planned through forthcoming wellness centers in India. There are additional studies showing beneficial effects of Yoga on FBS [48], PPBS [49–51], HbA1c [50, 51], total cholesterol, LDL [50, 51]. The analysis of the yoga protocols used in above said studies reveal the incorporation of some common and important postures in DYP, which seem to be important in managing the disease. It is also the possible that the beneficial effects of mind body techniques are sensitive to mental disposition of subjects and has been characterized by various measures like psychometric analysis [52, 53], namely, *Tridosha* and *Triguna* scoring [54, 55]. These were not analyzed in this study.

Briefly, DYP’s promising efficacy on glycemic and metabolic parameters requires mechanistic insights. This can be examined by further studies, and long term follow up which was not possible in this study. As DYP is a non-pharmacological, cost-effective method to halt the conversion of early diabetes into prediabetes and/or healthy individuals, the success of its integration into public health policy will depend on its wider acceptability and perception of benefits by both public as well as healthcare workers [56–59]. Yoga’s benefits in maintaining and regulation of the glycemic status are supported by several other studies [49, 50], which might enable its inclusion in the National *Ayushman Bharat* scheme or as part COVID pandemic management protocol in which a large number of individuals with diabetes and heart disease are falling prey [60, 61]. This will further encourage molecular and Ayurgenomic studies which presumably underlie the stated clinical outcome.

### Limitations

Moreover, there are some limitations of our study that we only studied in two regions of North India and thus the result of this study cannot be generalized on the remaining population. Further, in this study, the socio economic status and psychological assessments were not carried out. We were not able to control for the dietary habits and psychological status of the study participants. However, the small sample size and absence of long term evaluations limit the strength of the study.

## Conclusion

The findings indicate that the DYP intervention could improve the metabolic status of the high-diabetes-risk individuals with respect to their glucose tolerance and lipid levels, partially explained by the reduction in abdominal obesity. The study highlights the potential role of yoga intervention in real time improvement of cardiovascular profile in a high diabetes risk cohort.

## Data Availability

The datasets used during the present study are available from the corresponding author on reasonable request.

## Abbreviations

ADA: American Diabetes Association
BMI: Body mass Index
CVD: Cardiovascular disease
DYP: Diabetic yoga protocol
FBS: Fasting blood sugar
HbA1c: Glycated hemoglobin
HDL-c: High density lipid-cholesterol
IDRS: Indian Diabetes Risk Score
IFG: Impaired fasting glucose
IGT: Impaired glucose tolerance
IYA: Indian Yoga Association
LDL-c: Low density lipid-cholesterol
NMB: *Niyantarita Maduhmeha Bharat*
OGTT: Oral glucose tolerance test
PPBG: Postprandial blood glucose
SBP: Systolic blood pressure
VLDL: Very low density lipid-cholesterol
WC: Waist circumference
YVDM: Yoga volunteers for diabetes management
